# Aerosol emission and superemission during human speech increase with voice loudness

**DOI:** 10.1038/s41598-019-38808-z

**Published:** 2019-02-20

**Authors:** Sima Asadi, Anthony S. Wexler, Christopher D. Cappa, Santiago Barreda, Nicole M. Bouvier, William D. Ristenpart

**Affiliations:** 1 Department of Chemical Engineering, University of California Davis, 1 Shields Ave, Davis, CA 95616 USA; 2 Department of Mechanical and Aerospace Engineering, University of California Davis, 1 Shields Ave, Davis, CA 95616 USA; 3 Air Quality Research Center, University of California Davis, 1 Shields Ave, Davis, CA 95616 USA; 4 Department of Civil and Environmental Engineering, University of California Davis, 1 Shields Ave, Davis, CA 95616 USA; 5 Department of Land, Air and Water Resources, University of California Davis, 1 Shields Ave, Davis, CA, 95616 USA; 6 Department of Linguistics, University of California Davis, 1 Shields Ave, Davis, CA 95616 USA; 7 Department of Medicine, Div. of Infectious Diseases, Icahn School of Medicine at Mount Sinai, 1 Gustave Levy Place, New York, NY 10029 USA; 8 Department of Microbiology, Icahn School of Medicine at Mount Sinai, 1 Gustave Levy Place, New York, NY 10029 USA

## Abstract

Mechanistic hypotheses about airborne infectious disease transmission have traditionally emphasized the role of coughing and sneezing, which are dramatic expiratory events that yield both easily visible droplets and large quantities of particles too small to see by eye. Nonetheless, it has long been known that normal speech also yields large quantities of particles that are too small to see by eye, but are large enough to carry a variety of communicable respiratory pathogens. Here we show that the rate of particle emission during normal human speech is positively correlated with the loudness (amplitude) of vocalization, ranging from approximately 1 to 50 particles per second (0.06 to 3 particles per cm^3^) for low to high amplitudes, regardless of the language spoken (English, Spanish, Mandarin, or Arabic). Furthermore, a small fraction of individuals behaves as “speech superemitters,” consistently releasing an order of magnitude more particles than their peers. Our data demonstrate that the phenomenon of speech superemission cannot be fully explained either by the phonic structures or the amplitude of the speech. These results suggest that other unknown physiological factors, varying dramatically among individuals, could affect the probability of respiratory infectious disease transmission, and also help explain the existence of superspreaders who are disproportionately responsible for outbreaks of airborne infectious disease.

## Introduction

It has long been recognized that particles expelled during human expiratory events, such as sneezing, coughing, talking, and breathing, serve as vehicles for respiratory pathogen transmission^1–6^. The relative contribution of each expiratory activity in transmitting infectious microorganisms, however, remains unclear^4^. Much previous research has focused on coughing^7–12^ and sneezing^11,13,14^ activities that yield relatively large droplets (approximately 50 μm or larger) easily visible to the naked eye. Less noticeable, but arguably more infectious for some diseases, are the smaller particles emitted during sneezing and coughing as well as during breathing^15–17^ and talking^16,18,19^. These small particles are believed to be generated during breathing and talking from the mucosal layers coating the respiratory tract via a combination of a “fluid-film burst” mechanism within the bronchioles and from vocal folds adduction and vibration within the larynx^6,20,21^. The particles emitted during breathing and typical speech predominantly average only 1 μm in diameter^15–17^ and are thus too small to see without specialized equipment; most people outside of the community of bioaerosol researchers are less aware of them.

Despite their small size, however, these micron-scale particles are sufficiently large to carry a variety of respiratory pathogens such as measles virus (50–500 nm)^22^, influenza virus (100 nm–1 µm)^23^, and *Mycobacterium tuberculosis* (1–3 µm)^24^. Indeed, recent work by Yan *et al*. has confirmed that significant amounts of influenza viral RNA are present in small particles (<5 μm) emitted by influenza-infected individuals during natural breathing, without coughing or sneezing^25^. These small particles are potentially more infectious than larger sneeze- or cough-generated droplets for several reasons. First, smaller particles persist in the air for longer time periods before setting by gravity, thus increasing the probability of inhalation by susceptible individuals^26^. Second, smaller particles have a larger probability of penetrating further into the respiratory tract of a susceptible individual to initiate a lower respiratory tract infection^4^. Third, and perhaps most importantly, speech can release dramatically larger numbers of particles compared to coughing. Early work by Papineni and Rosenthal^16^ and Loudon and Roberts^19^ reported that speaking (as exemplified by counting aloud) releases about 2–10 times as many total particles as a single cough. Similarly, Loudon and Roberts investigated the role of singing in the spread of tuberculosis and showed that the percentage of airborne droplet nuclei generated by singing is 6 times more than that emitted during normal talking and approximately equivalent to that released by coughing^27^. More recent work using advanced particle characterization techniques have yielded similar results^21,28–30^. Chao *et al*.^28^ used an interferometric imaging technique to obtain the size distribution of particles larger than 2 μm and found that counting aloud from 1 to 100 releases at least 6 times as many particles as an individual cough. Likewise, Morawska and coworkers^21,29^ reported that counting aloud for 10 seconds followed by 10 seconds of breathing, repeated over two minutes, releases half as many particles as 30 seconds of continual coughing, which in turn releases half as many particles as saying “aah” for 30 seconds. They also reported that more particles are released when speech is voiced, which involves vocal folds vibration, rather than whispered, which does not.

Despite the clear evidence that speech emits large quantities of potentially infectious particles, to date little is known about how particle emission is modulated by different types of speech. Notably, the above work measured neither the total duration nor the loudness of the vocalizations; it is also unclear whether counting aloud will have a distribution of phones (phonemes) that is representative of typical conversational speech. Many important questions remain unanswered. For example, does raising your voice cause an increase in particle emission, or alter the particle size distribution? Does it matter what language you speak? Do all individuals emit particles at similar rates?

To address these questions, we used an aerodynamic particle sizer (APS) placed in a laminar flow hood to characterize the number and size distribution of particles emitted by individual human volunteers while they performed various vocalizations and breathing activities. Using this approach, we find three key results:

1. The particle emission rate during speech is linearly correlated with the amplitude (loudness) of vocalization, for four different languages tested.
2. The particle size distribution is independent of vocalization loudness or language spoken.
3. Some individuals emit particles at a rate more than an order of magnitude larger than their peers, i.e., they behave as “speech superemitters.”

Taken together, the results strongly suggest that individual human speech patterns and speech-associated particle emissions are highly heterogeneous and thus might play a role in the transmission of some respiratory pathogens. Furthermore, the results suggest a new hypothesis: that speech superemitters might contribute to the phenomenon of superspreading, in which a relative few contagious individuals infect a disproportionately large number of secondary cases during infectious disease outbreaks^31^.

## Results

**Figure 1:**
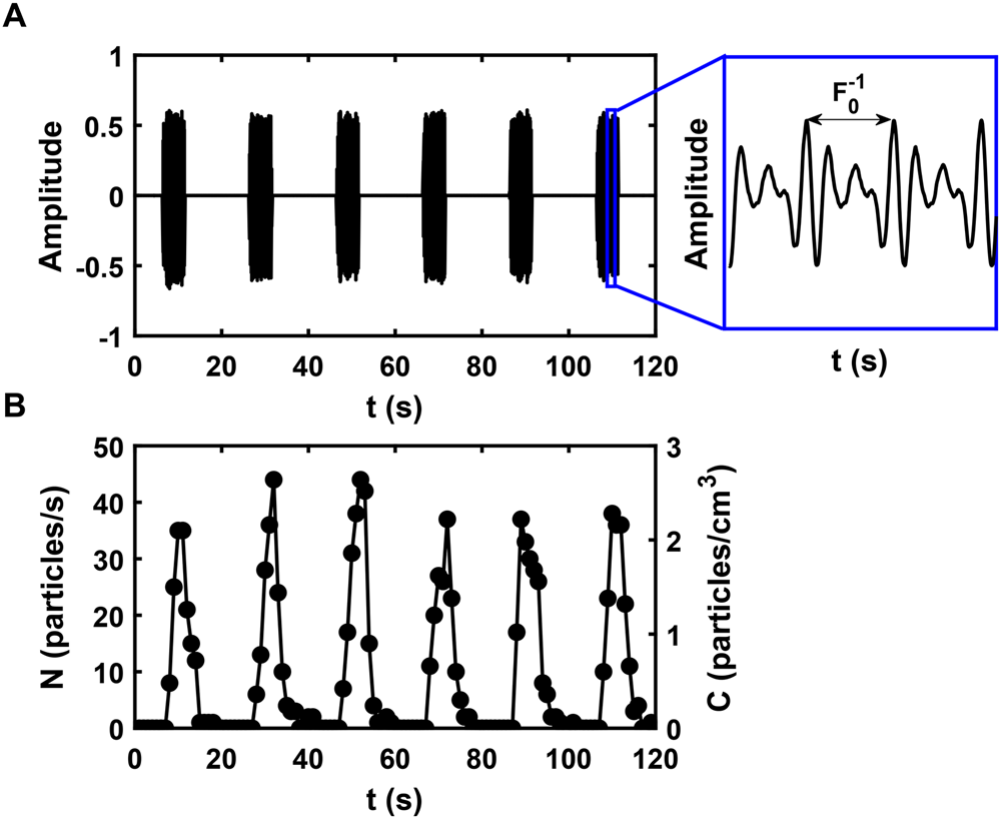
Representative raw data in which a participant (F4) said /ɑ/ for 5 seconds, followed by 15 seconds of nose breathing, repeated 6 times at approximately the same loudness. (**A**) The amplitude (arb. units) recorded by the microphone versus time. Magnification shows 13 ms of the waveform with fundamental frequency of F_0_. (**B**) The corresponding number/concentration of particles measured by the APS versus time.

Four separate types of experiments were performed. In the first experiment, participants said /ɑ/ (the vowel sound in ‘saw’) for five seconds, followed by 15 seconds of nose breathing, repeated six times in succession. This procedure mimics previous experimental measurements of particle emission during vocalization^21^, but here the participants also systematically repeated the experiment at different voice amplitudes. Representative raw data for a single participant performing a series of six successive /ɑ/ vocalizations, at approximately the same loudness, are shown in Fig. 1. The simultaneous microphone recording (Fig. 1A) and APS measurements (Fig. 1B) demonstrate that the dynamics of particle release are highly correlated with the vocalization. Prior to and between vocalizations, during nose breathing in which exhaled air is directed away from the APS, the particle count is negligible, as is expected for the HEPA filtered air inside the laminar flow hood. Shortly after the vocalization commences, the number of particles rapidly increases and peaks, then decreases back to zero as the participant resumes nose breathing; the process then repeats at the next five-second vocalization. The approximately two-second lag between onset of vocalization and the observed increase in particle count is due to the time necessary for the released particles to reach the sensor in the APS. We emphasize that by design an APS does not measure 100% of the particles drawn into it, so the particle emission rates reported here do not represent the absolute number of particles emitted by the participant; the emission rates are best understood in relative terms, or in terms of the equivalent instantaneous concentrations of particles sampled from the funnel. As shown in the secondary axis of Fig. 1B, the instantaneous concentration of particles for this particular experiment was approximately 2 per cm^3^ of sampled air.

**Figure 2:**
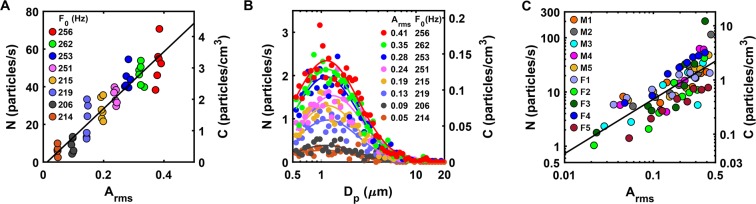
Particle emission rate/concentration while saying /ɑ/ at 8 different amplitudes, repeated 6 times at each amplitude. (**A**) Particle emission rate/concentration versus root mean square amplitude, A_rms_ (arb. units) for a representative participant (F4). Solid line is the best linear fit, with correlation coefficient ρ = 0.932 and Pearson’s p value = 5.9 × 10^−22^. (**B**) Corresponding particle size distribution for the data presented in (**A**). (**C**) Aggregated particle emission rate/concentration versus root mean square amplitude, A_rms_ (arb. units) for 10 participants, 5 males (denoted as M1 to M5) and 5 females (denoted as F1 to F5). There are 8 data points for each participant, each representing the average of repeating /ɑ/ six times at approximately the same voice amplitude (cf. Fig. 1). Solid line is a power law fit with exponent 1.004, correlation coefficient ρ = 0.774 and Pearson’s p value = 3.8 × 10^−17^.

The six vocalizations shown in Fig. 1A were made, to the best of the participant’s ability, at the same loudness. Each participant then repeated a similar series of /ɑ/ vocalizations at different self-regulated voice amplitudes. Representative results for a single participant (F4) show that the particle emission rate (N), defined as the total number of particles emitted during a single vocalization divided by the measured duration (in seconds) of that vocalization, also correlates with the root mean square amplitude (A_rms_) of the vocalization (Fig. 2A). In our set-up A_rms_ = 0.45 corresponds to an extremely loud conversational voice, as loud as comfortable without yelling (~98 decibels measured 6.5 cm from the participant’s mouth, measured over background noise of approximately 65 decibels), while A_rms_ = 0.02 corresponds to a quiet vocalization just above whispering (~70 decibels; cf. Supplementary Fig. S1). As shown in Fig. 2A, the particle emission rate is linearly correlated with A_rms_ over this entire range of vocalization amplitudes, with the particle emission rate increasing from 6 to 53 particles per second at the quietest and loudest vocalizations respectively.

Although the particle emission rate increased with amplitude, the size distribution of the particles was not affected significantly (Fig. 2B), with the geometric mean particle diameter remaining near 1 μm regardless of voice amplitude (Supplementary Fig. S2A). Because the particle size remains similar regardless of amplitude, the increased particle counts shown in Fig. 2 indicate that the total volume of emitted respiratory fluid (i.e., the proteinaceous liquid droplets aerosolized from the serous and mucoid layers lining the respiratory tract) increases considerably with the vocalization loudness. Note that the characteristic time scale for evaporative drying of 1-micron diameter droplets is on the order of 100 milliseconds^26^, which is much less than the time required for the particles to move from the participant’s mouth into the detection module within the APS, suggesting that the particles measured here had fully dried into droplet nuclei prior to measurement (see methods and Supplementary Fig. S3).

Experiments with multiple participants indicated that these trends are conserved over a larger sample size (Fig. 2C). The particle emission rate increased approximately linearly with A_rms_ for each of the study participants, although the absolute magnitude varied between individuals. One participant (F3) released as many as 200 particles per second at higher amplitudes; another (F2) released as few as 1 particle per second at lower amplitudes. Notably, the data with this cohort of non-elderly adults reveal no obvious trends with gender or age (Supplementary Figs S4A, B). Similarly, no clear correlation was observed with the body mass index (BMI) of the participants (Supplementary Figs S4C, D).

**Figure 3:**
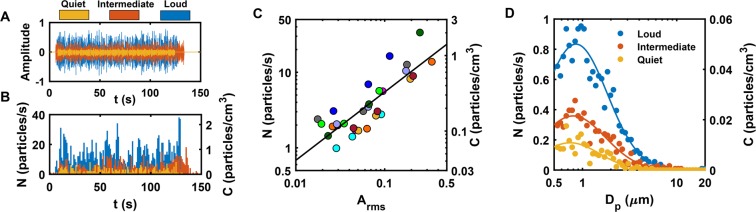
Particle emission rate/concentration while reading a passage of text aloud (the “Rainbow” passage), at three different loudness levels. (**A**) Superimposed representative recordings of amplitude (arb. units) for an individual (F4) reading the passage at three different voice amplitudes, and (**B**) the corresponding number/concentration of particles measured by the APS versus time. Color code same as in (**A**). (**C**) Particle emission rate/concentration as a function of root mean square amplitude, A_rms_, for 10 participants. There are 3 points for each person, representing 3 voice amplitudes, color code same as Fig. 2C. Solid line is a power law fit with exponent 0.96, correlation coefficient ρ = 0.865 and Pearson’s p value = 6.8 × 10^−10^. (**D**) Representative particle size distribution for the one individual (F4).

To more closely represent normal conversational speech, the participants read aloud a short passage of text in English at varied loudness (quiet, intermediate, or loud). Representative raw data for a single participant (F4) indicate that the particle emission rate also correlates with voice amplitude for normal speech (Fig. 3A,B). To quantify the loudness, we take A_rms_ here as the average over the entire approximately two-minute duration of the vocalization, excluding pauses between words. Aggregated data for 10 participants confirms that the particle emission rate for normal English speech correlates linearly with A_rms_ (Fig. 3C); speaking loudly yielded on average a 10-fold increase in the emission rate compared to speaking the same series of words quietly. Again, the size distributions (Fig. 3D) and geometric mean diameter of particles (Supplementary Fig. S2B) were insensitive to voice amplitude. The reading experiment also was repeated in different languages to test whether choice of language matters; the results (Supplementary Fig. S5) confirmed the increasing trend between particle emission rate and amplitude, but exhibited no significant difference in the particle emission rate among the languages tested (Supplementary Fig. S6). Likewise, we measured the temperature and humidity during the experiments, and found no significant impact of temperature or humidity on either the particle emission rate or the mean particle size (Supplementary Figs S7 and S8).

**Figure 4:**
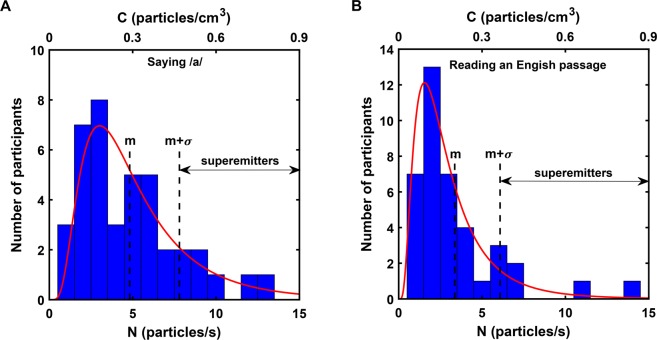
Histogram of particle emission rate/concentration at voice amplitude of 0.1 (approximately 85 dB). (**A**) For saying /ɑ/, with median of M = 4.3 particles/s, mean of m = 4.8 particles/s and standard deviation of σ = 3.0 particles/s. (**B**) For reading an English passage (10 people read the “Rainbow” passage and 30 people read chapter 24 of “The Little Prince”) with median of M = 2.5 particles/s, mean of m = 3.4 particles/s and standard deviation of σ = 2.7 particles/s. Particle emission rates larger than m + σ are labeled superemitters. Red curves are lognormal fits found via nonlinear regression.

A key recurring feature of the data is that some individual participants emitted many more particles than others. Because all participants spoke at slightly different amplitudes, we used linear regressions of the particle emission rate versus amplitude for each individual (cf. Fig. 2A) to calculate a normalized particle emission rate at the loudness amplitude of 0.1 (approximately 85 dB). Using this approach, the results for 40 people show that the particle emission rate for different individuals follows a long-tailed distribution for both vocalization of /ɑ/ (Fig. 4A) and reading of English text aloud (Fig. 4B). At this loudness, the normalized particle emission rates ranged from approximately 1 to 14 particles per second between different individuals, with an average of approximately 4 particles per second. Notably, the rates have a sizeable standard deviation well approximated by a lognormal fit (red curves in Fig. 4). In other words, although half of the participants emitted fewer than 3 particles per second, a small fraction of individuals (8 out of 40) emitted considerably more. These “speech superemitters,” whose individual particle emission rate exceeded the group mean by one standard deviation or more, consistently released an order of magnitude more particles than their peers. For vocalizing /ɑ/, Fig. 4A shows that 15% of the participants emitted 32% of the total particles, while Fig. 4B shows that, for reading aloud in English, 12.5% of the participants emitted 40% of the total particles. Supplementary Fig. S9A shows that 4 out of these 8 individuals are superemitters for both saying /ɑ/ and passage reading activities, while 2 of them are only superemitters while saying /ɑ/, and 2 of them are superemitters while reading a text passage. We repeated the passage reading experiment for two of the participants (M5 and F4) on three different days separated by several months (Supplementary Fig. S9B), and the results show that the particle emission rates remained almost unchanged for at least these two individuals (F4, a superemitter, and M5, a non-superemitter) despite the long time period between measurements.

**Figure 5:**
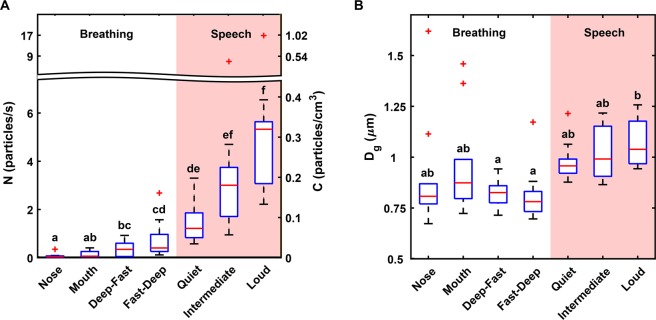
Comparison of (**A**) emission rate/concentration and (**B**) corresponding geometric mean diameters of particles emitted during various modes of breathing versus speech at different loudness levels. “Nose” denotes normal nasal breathing; “Mouth” denotes normal mouth breathing; “Deep-Fast” denotes deep, slow nasal inhalation followed by fast mouth exhalation; “Fast-Deep” denotes fast nasal inhalation followed by deep (i.e., slow and prolonged) mouth exhalation. “Quiet”, “Intermediate”, and “Loud” denote loudness levels while reading aloud a passage of text (“Rainbow” passage) at respective amplitudes. Red lines indicate medians, while bottom and top of blue boxes indicate the 25^th^ and 75^th^ percentiles respectively; sample size is n = 10. Outliers (defined as values that exceed 2.7 standard deviations) are indicated with red plus signs. Note that the 2 outliers for speech in (**A**) are a different individual (F4) than the two outliers observed for nose and fast-deep breathing (M24 and M5 respectively). Scheffe groups are indicated with letters; groups with no common letter are considered significantly different with p < 0.05, cf. Supplementary Table S1. Note that (**A**) has different scales above and below the break.

To help interpret our findings we also compared the particle emission rates of four different types of breathing with speech at three levels of loudness using the same experimental set-up. The breathing experiments included nose breathing, mouth breathing, a “deep-fast” mode, and a “fast-deep” mode (see methods for details). The results show that the particle emission rate for speech is significantly higher than all types of breathing tested here (Fig. 5A). Furthermore, the corresponding geometric mean diameters of the particles generated during speech are slightly larger on average than those generated during breathing (Fig. 5B), consistent with prior work and the hypothesis that vocalization activates laryngeal particle generation^21^. Note that in Fig. 5A the speech outliers correspond to a single participant who is a speech superemitter (F4), but this individual was not also responsible for the observed outliers of “fast-deep” and “nose” breathing activities. In other words, the “breathing high producers” as defined by Edwards *et al*.^15^ are not necessarily also speech superemitters.

## Discussion

Given that the results clearly indicate that particle emission rate is correlated with vocalization amplitude, a natural question is: why? The particles emitted during breathing and speech are hypothesized to be formed primarily by a “fluid-film burst” mechanism inside the small airways of the lungs and/or via vocal folds vibration and adduction at the larynx^6,20,21^. During exhalation the elastic walls of the respiratory bronchioles contract, and the mucosal fluid on the lumen surface forms a continuous film that can completely fill the airway. During the subsequent inhalation, the bronchioles expand and the film ruptures, yielding particles that are drawn into the alveoli and subsequently exhaled. A similar mechanism is believed to occur in the larynx, as the vocal folds repeatedly close and open during vocalization^21^; when the vocal folds come into contact during adduction, fluid films that form between them can then rupture during their subsequent abduction. Our direct comparison of particles emitted during various types of breathing versus speech demonstrates that even quiet speech yields significantly more particles than normal breathing (Fig. 5A). Coupled with the observation that the particles generated during speech on average are slightly larger (Fig. 5B), the results suggest that laryngeal particle generation, which presumably does not occur during normal breathing, is at least partially responsible for the observed larger rates of particle emission. Indeed, the fundamental frequency or “pitch” of vocalization (i.e., the frequency at which the vocal folds open and close) increases slightly with amplitude (cf. Supplementary Fig. S11 and Gramming *et al*.^32^), so the increased amplitude could reflect an increased opportunity for particles to form at the larynx.

Complicating matters, however, vocalization at a larger voice amplitude requires a larger exhalation flow rate^33,34^. A possible interpretation of our observations is that the underlying physical mechanism of particle release hinges on the combination of laryngeal particle generation rate and the time integral of the exhalation flow rate during vocalization^35^. If the volume of exhaled air is larger when the voice amplitude is higher, a larger fraction of particles formed in bronchiolar film rupture may escape from the lungs, with consequently more emitted particles, thus increasing the particle concentration in the exhaled air. Since our measurements only gauge the particle emission rate (and equivalent concentration), it is difficult to decouple the relative contributions of these two mechanisms. Fitting our particle size distributions to constrained bimodal lognormal distributions provides some evidence consistent with the interpretation presented by Johnson *et al*.^21^ that there are two modes, presumably due to bronchiolar versus laryngeal generation, but we do not find any significant difference in particle emission rates for the two modes as a function of vocalization amplitude (Supplementary Fig. S10 and cf. Fig. 5B). Furthermore, it is less understood how particles originating in the respiratory tract might deposit in more proximal regions instead of being emitted during exhalation. Particle deposition efficiency during nasal exhalation is known to depend on exhalation flow rate in a convoluted fashion, with Brownian diffusion, sedimentation, and inertial impaction all playing roles at different length and time scales within the respiratory tract^36^. Nonetheless, our results strongly suggest that, in general, more particles escape the respiratory tract if the vocalization is louder.

Our results also clearly show that some participants release many more particles than others, for as-yet unclear reasons. It is known that the Rayleigh-Plateau instability that gives rise to small droplets during the “film burst” is sensitive to the interfacial tension, density, and viscosity of the fluid^37,^ so one possible explanation is that the mucosal fluids in different people have different material properties and correspondingly generate more or fewer drops. Notably, different disease states are known to alter the physicochemical properties of the mucosal fluid lining the respiratory tract^38^, so it is possible that infected individuals might generate markedly different quantities of particles than those emitted by the healthy individuals tested here. Intriguingly, Edwards *et al*.^15^ found that delivering nebulized isotonic saline to individuals decreased the number of particles exhaled during normal breathing for a few hours after inhalation of the saline; further tests are warranted with speech. Alternatively, it is possible that individual manners of articulation affect the amount of internal deposition of the particles before they manage to escape the mouth. Our tests of different languages yielded no significant differences, at odds with previous speculation that language spoken might have played a role in the epidemiology of SARS coronavirus transmission^39^, and suggesting that some as yet unknown physiological factor causes the dramatic variation among individuals.

Regardless of the underlying physical mechanism, from an epidemiological perspective the existence of speech superemitters motivates consideration of a new hypothesis: that speech superemitters contribute to “superspreading” of infectious diseases transmitted by emitted airborne particles. A superspreader is a contagious individual who infects a disproportionately large number of susceptible contacts^31,40,41^. To date, several airborne superspreading events have been documented, such as the MERS-CoV outbreak in South Korea in 2015 and the SARS-CoV outbreak in 2003, the latter being initiated in Hong Kong and spreading to Canada, Vietnam, and Singapore through travel^40–43^. In the case of respiratory infectious diseases in particular, the underlying physiological and immunological factors that contribute to heterogeneity in individual infectiousness remain poorly understood, despite the epidemiological importance of respiratory superspreaders. Quantifying infectious pathogen loads in exhaled air is technically challenging, relative to other contagious substances like blood, urine, and feces. Many factors presumably affect the secondary attack rate attributable to any infectious individual, including the herd immunity status of others in proximity. Nonetheless, our results suggest that, for respiratory infections transmitted from person to person via airborne particles, the existence of speech superemitters might help explain the existence of superspreaders. A similar hypothesis was advanced by Edwards *et al*.^15^ in response to their observation of variability between individuals in the number of particles emitted during mouth breathing. Interestingly, our data show that speech superemitters are not necessarily breathing superemitters as well (Fig. 5A), suggesting that respiratory superemission during vocalized speech has a different underlying physiology than superemission during tidal breathing.

Our results indicate that speech is potentially of much greater concern than breathing for two reasons: the particles on average are larger, and thus could potentially carry a larger number of pathogens, and much greater quantities of particles are emitted compared to breathing, thus increasing the odds of infecting nearby susceptible individuals. Laryngeal particle generation during speech is also potentially important since some studies suggest that human influenza viruses attach more abundantly to the large airways of the upper respiratory tract than to the bronchiolar and alveolar cells in the lower respiratory tract, while MERS-CoV and avian influenza viruses mainly cause lower respiratory tract infections due to the greater presence of these virus receptors deeper within the lung^44–47^; likewise there is evidence that laryngeal tuberculosis is potentially more contagious than typical pulmonary tuberculosis^48^.

A second key epidemiological implication of our results is that simply talking in a loud voice would increase the rate at which an infected individual releases pathogen-laden particles into the air, which in turn would increase the probability of transmission to susceptible individuals nearby^49^. For example, an airborne infectious disease might spread more efficiently in a school cafeteria than a library, or in a noisy hospital waiting room than a quiet ward. Moreover, our data suggest a related hypothesis, that infected individuals could be transmitting significant numbers of respiratory pathogens via speech in the absence of overt clinical signs of illness like coughing or sneezing. More research is needed; however, the presence of asymptomatic or paucisymptomatic superspreaders would have important public health implications in the surveillance for and mitigation of infectious disease epidemics that are spread by airborne respiratory particles. The data presented here strongly suggest that further efforts to test these hypotheses are warranted.

## Methods

### Human subjects

The University of California Davis Institutional Review Board approved this study and all research was performed in accordance with relevant guidelines and regulations of the Institutional Review Board. We recruited 48 healthy volunteers (26 males and 22 females, ranging in age from 18 to 45 years old) by posting flyers at the University of California Davis campus over the time period May 2016 to March 2018. Informed consent was obtained from all participants prior to study participation. All participants completed a brief questionnaire including age, gender, weight, height, general health status, and smoking history. Only participants who self-reported as healthy non-smokers were included in the study. The subject in Supplementary Fig. S12 provided her written informed consent for the publication of identifying information/images in an online open-access publication.

### Experimental set-up

A photograph of the experimental set-up is provided as Supplementary Fig. S12. An aerodynamic particle sizer (APS, TSI model 3321) operating at a total flow rate of 5 L/min (sheath flow rate ≅ 4 L/min, sample flow rate ≅ 1 L/min) was placed inside a HEPA filtered laminar flow hood that provided class 10 air. A plastic funnel (diameter = 10 cm) was connected to the APS sampling inlet via a conductive silicon tube (distance between funnel hole to APS inlet = 7.5 cm, tube inner diameter = 1.2 cm). During each experiment, participants sat at the laminar flow hood, in front of the APS, and spoke into the funnel. For the majority of speaking and breathing experiments, a nose rest across the funnel opening was used to position participants’ mouths approximately 7.5 cm away from the funnel inlet (hole) and also to divert nasal exhalations away from the APS. During “nose-breathing” experiments, the nose rest was removed to allow nasal exhalations to be drawn into the APS. Note that participants’ faces did not touch the funnel, so that air was free to move around the side of their faces; in this sense the cone was a semi-confined environment and not all expired particles were necessarily sampled by the APS. Also note that the sheath flow inside of an APS is filtered, so the particle emission rates sampled by the APS automatically remove 80% of the particles sampled from the funnel. Equivalent concentrations reported on the secondary axes in Figs 1 through 5 are determined from the raw particle counts using the sample flow rate, i.e., ${\rm{C}}=\frac{{\rm{particles}}}{{\rm{s}}}\times \frac{{\rm{s}}}{{{\rm{cm}}}^{3}}=\frac{{\rm{particles}}}{{{\rm{cm}}}^{3}}$. Also note that the APS measures the size distribution of particles larger than 0.5 µm, but only detects the presence of particles between 0.37 µm and 0.5 µm without providing precise size measurements. For this reason Figs 1–5 exclude the counts of particles smaller than 0.5 µm; including them has little impact on the results since the vast majority of particles were larger than 0.5 microns.

A microphone (audio-technica PRO 37) and a decibel meter (Extech, 407760) were placed immediately on either side of the funnel to record the vocalizations. A computer screen with word prompts and a timer was placed behind the APS to guide participants in making requested vocalizations for the specified duration. The timing, duration, repetition, and order of vocalization and breathing experiments were coordinated by customized code written in LabVIEW (National Instruments). A digital hygrometer was used to measure the ambient temperature and relative humidity inside the laminar flow hood during all experiments. The participants were not allowed to drink or eat during the experiment, but they were free to rest between experiments for a few minutes as needed; data from each individual participant was gathered over an approximately 1-hour time period. We performed the experiments in an indoor (controlled) environment, so the ambient temperature varied only from approximately 20 to 25 °C, while the ambient relative humidity measured inside the laminar flow hood varied from a low of approximately 45% to a high of 80%. Control experiments indicate that the particle size distribution was independent of whether the particles were expired early or late during a sustained vocalization (Supplementary Fig. S3), indicating that transient fluctuations in the humidity inside the funnel due to exhalation had no impact on the final measured size distribution. Particles with initial diameter of less than 20 µm dry to approximately half of their initial diameter in less than 1 second^49,50^. Different correction factors have been suggested in the literature that one can use to estimate the initial size of the particles^49,51^; here we focus on the final size distribution because epidemiologically it is the final size distribution governs the deposition efficiency of the particles in the respiratory tract of nearby susceptible individuals^52^.

### Vocalization experiments

#### “/ɑ/” experiments

Participants (n = 10, 5 males, M1 to M5, and 5 females, F1 to F5) voiced /ɑ/ (the vowel sound in ‘saw’) for five seconds, followed by 15 seconds of nose breathing, repeated six times in succession. The participant repeated the series of six /ɑ/ vocalizations, to the best of the participant’s ability, at the same amplitude. Each participant completed eight sets of /ɑ/ experiments, each set performed at different, self-regulated voice amplitude. Timed prompts with directions for the requested vocalization appeared on the computer screen, which displayed a timer and an amplitude (loudness) gauge to help the participants regulate their voice amplitude. The requested amplitudes were presented to participants in a random order.

#### “Rainbow passage” experiments

Participants (n = 10, 5 males, M1 to M5, and 5 females, F1 to F5) read aloud a 330-word excerpt of text in English, known in linguistics research as the Rainbow passage^53^. Participants were asked to read the Rainbow passage aloud three times, at a comfortable pace, over approximately 2 minutes per reading. Each of the three readings was performed at a different self-regulated amplitude: quiet, intermediate, and loud. Quiet was defined for participants as “just louder than a whisper,” intermediate as a “normal conversational voice,” and loud as “giving a loud lecture”.

#### “The Little Prince” experiments

Bilingual participants (n = 30) fluent in both English and either Spanish (n = 10, 5 males, M6 to M10, and 5 females, F6 to F10), Mandarin (n = 10, 5 males, M11 to M15, and 5 females, F11 to F15), or Arabic (n = 10, 6 males, M16 to M21, and 4 females, F16 to F19) read Chapter 24 of “The Little Prince^54^” aloud six times, three times in English translation, each time at a different amplitude (quiet, intermediate, and loud) and three times in their respective language, again at three loudness levels.

#### Breathing/speaking experiments

Participants (n = 10, 6 males, M5 and M22 to M26, and 4 females, F4 and F20 to F22) alternated four silent breathing patterns with vocalized speech at three amplitudes. For breathing measurements, the breathing patterns were designated as “nose” (both inhalation and exhalation through the nose), “mouth” (both inhalation and exhalation through the mouth), “deep-fast” (deep, slow inhalation for ~3 seconds through the nose, holding it for ~1 second, followed by fast exhalation through the mouth (~1 second)), and “fast-deep” (rapid inhalation through the nose (~1 second), holding it for ~1 second, followed by slow exhalation through the mouth for ~3 seconds). Each breathing experiment was performed over 2 minutes, and at a comfortable pace for the participants. Between performing different breathing patterns, participants were asked to read the Rainbow passage in a “quiet,” “intermediate,” or “loud” voice, as prompted by the computer in random order.

### Statistical analysis

Data analysis was performed in MATLAB (MathWorks), with data fits performed as noted in figure legends. Pearson’s linear correlation coefficients and p values were calculated for linear fits. Lognormal fits were made via nonlinear regression, and median, mean, and standard deviation were calculated. Box-and-whisker plots show the median (red line), interquartile range (blue box), and range (black whiskers). To analyze the breathing/speaking experiments data presented in Fig. 5, Stata/SE 15.1 was used to perform general linear mixed model (GLMM) analysis to account for person-level correlations, and post hoc pairwise comparisons were performed and adjusted for multiple comparisons using Scheffe’s method.

## Supplementary information

Supplementary Info

## Data Availability

All relevant data are available from the corresponding authors upon request.
