# Supplementary material for: Aerosol emission and superemission during human speech increase with voice loudness
Source: Sci Rep. 2019 Feb 20;9:2348. doi: 10.1038/s41598-019-38808-z (PMC6382806; doi:10.1038/s41598-019-38808-z)
Supplement: Supplementary file 1 — Supplementary Info [file 41598_2019_38808_MOESM1_ESM.pdf]

Aerosol emission and superemission during human speech increase with voice loudness

Sima Asadi, Anthony S. Wexler, Christopher D. Cappa, Santiago Barreda, Nicole M. Bouvier, & William D. Ristenpart*

*Corresponding author: William D. Ristenpart, Dept. of Chemical Engineering, University of California Davis, 1 Shields Ave., Davis, CA 95616 USA, (530) 752-8780, wdristenpart@ucdavis.edu

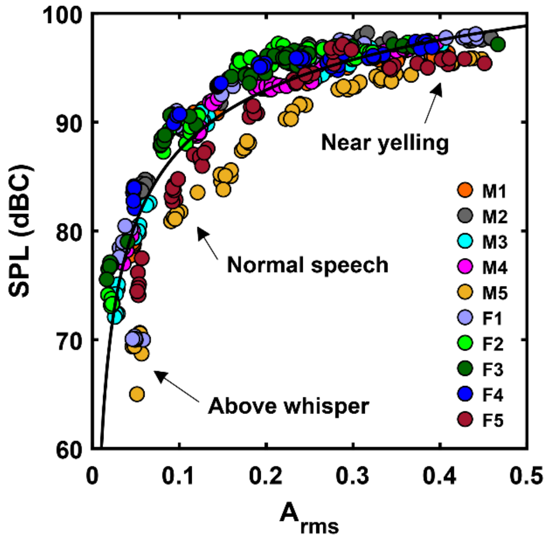

Supplementary Figure S1. Calibration curve relating the root mean square amplitude, A_{rms} (arb. units) recorded by the microphone with an independent measure of the sound pressure level, SPL, obtained from a decibel meter after removing the background noise, for saying /ɑ/ at 8 different amplitudes and 6 times at each amplitude for 10 people. Color codes identify individual participants (5 males denoted as M1 to M5 and 5 females denoted as F1 to F5). Black line is power law fit with exponent 0.094, correlation coefficient $\rho = 0.774$ and Pearson's p value = 3.6×10^{-17}.

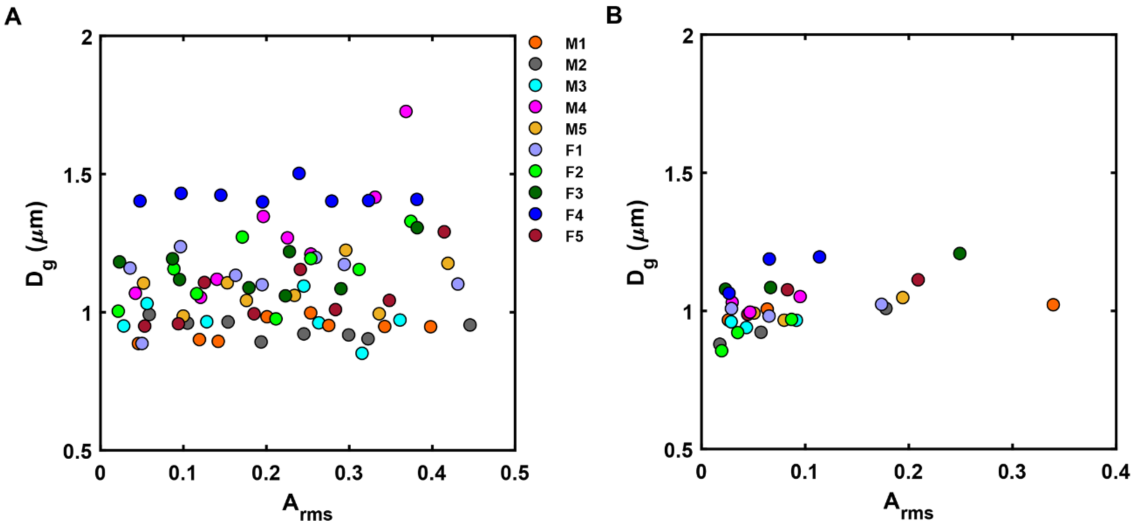

Supplementary Figure S2. Geometric mean diameter, D_g, for 10 people versus root mean square amplitude, A_{rms} (arb. units). Color codes identify individual participants. (**A**) Vocalizing /ɑ/ at 8 different amplitudes, (each data point is average of 6 times saying /ɑ/). (**B**) Reading aloud the “Rainbow” passage at 3 levels of loudness (quiet, intermediate, loud).

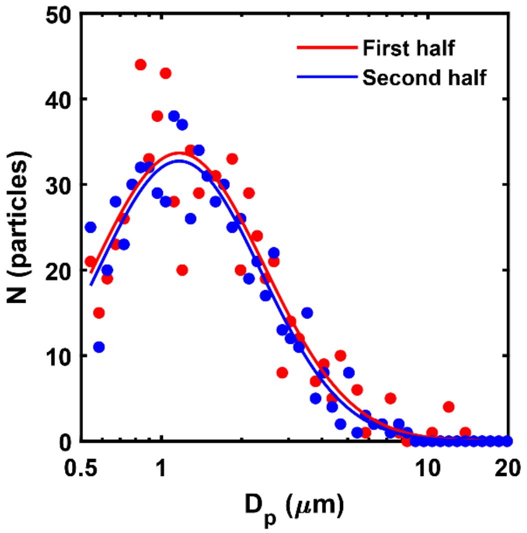

Supplementary Figure S3 – Size distribution of the particles released during the first half of a 5-sec vocalization (released to an initially dryer ambient air) repeated 6 times, compared to the size distribution of particles released during second half of the vocalization (released to moister ambient air). The size distribution is independent of when they were expired, indicating the particles have dried completely by the time they reach the APS sensor.

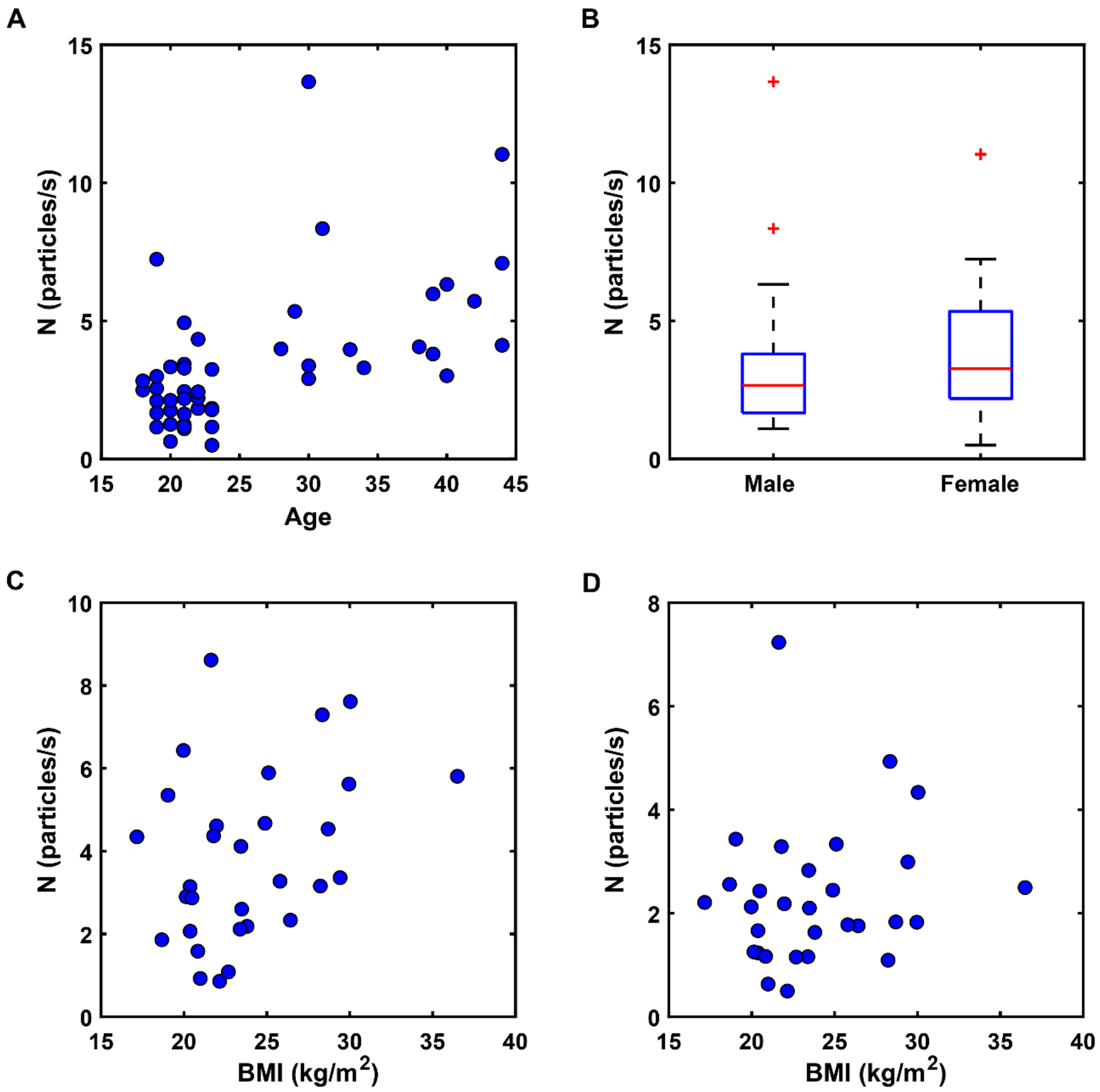

Supplementary Figure S4. Particle emission rate at voice amplitude of 0.1 (approximately 85 dB) for 48 people while reading and English passage, (**A**) versus age ($\rho = 0.55$, and $p = 4.9\times10^{-5}$), and (**B**) versus gender (Mann-Whitney U test p value = 0.26). Particle emission rate versus BMI at voice amplitude of 0.1 (approximately 85 dB) for 30 people, (**C**) while saying /ɑ/ ($\rho = 0.34$ and $p = 0.06$) (**D**) while reading an English passage ($\rho = 0.14$, and $p = 0.47$).

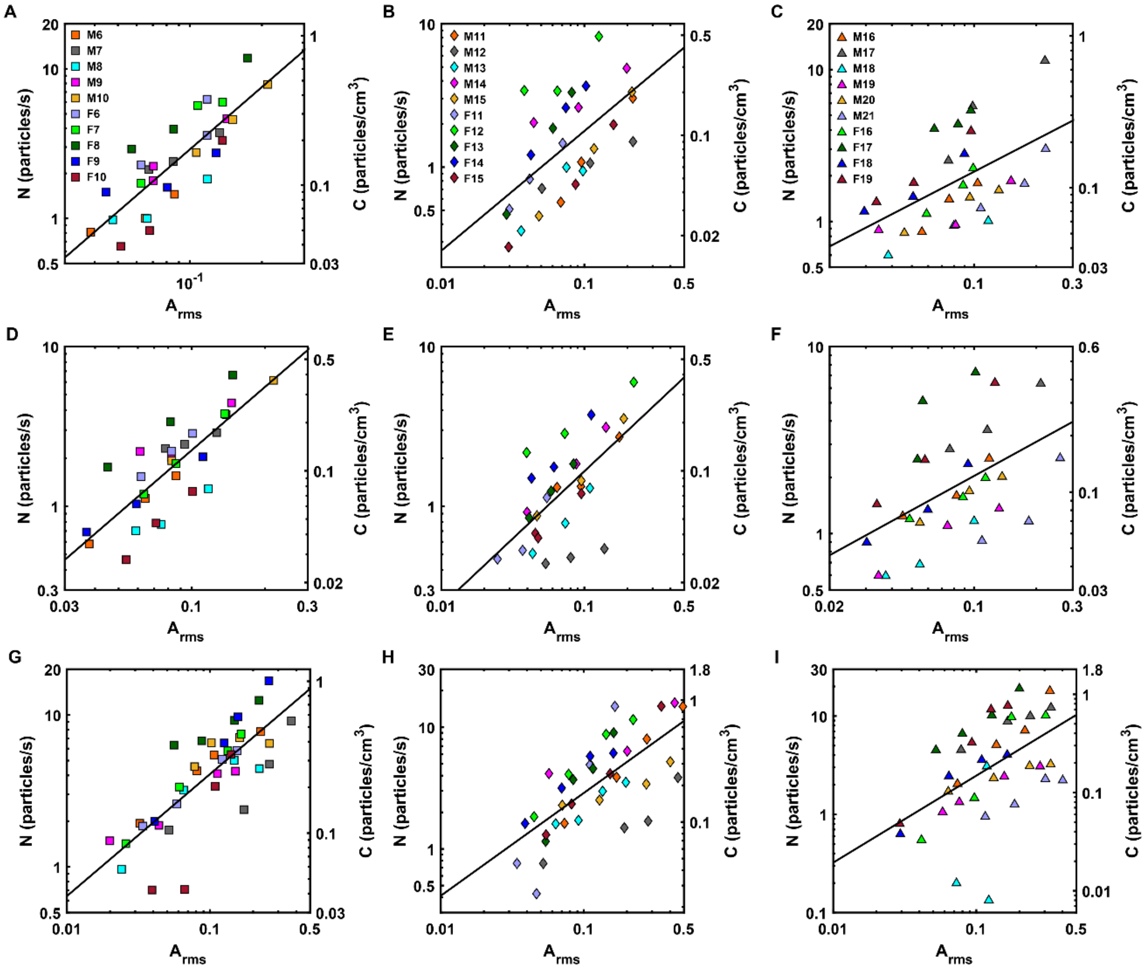

Supplementary Figure S5. Particle emission rate/concentration versus root mean square amplitude, A_{rms}, while reading chapter 24 of "The Little Prince" at three levels of loudness. Solid lines are power law fits with exponent n, correlation coefficient ρ, and Pearson's p value of p. (**A**) 10 Spanish speakers (5 males, 5 females, square markers) reading Spanish text ($n = 1.38$, $\rho = 0.82$, and $p = 2.1 \times 10^{-8}$), (**B**) 10 Mandarin speakers (5 males, 5 females, diamond markers) reading Mandarin text ($n = 0.84$, $\rho = 0.60$, and $p = 4.1 \times 10^{-4}$), (**C**) 10 Arabic speakers (6 males, 4 females, triangle markers) reading Arabic text ($n = 0.70$, $\rho = 0.54$, and $p = 2.2 \times 10^{-3}$). Particle emission rate/concentration for reading chapter 24 of "The Little Prince" in English at three levels of loudness by (**D**) 10 Spanish speakers ($n = 1.31$, $\rho = 0.84$, and $p = 4.8 \times 10^{-9}$, color code same as (**A**)), (**E**) 10 Mandarin Speakers ($n = 0.84$, $\rho = 0.76$, $p = 1.1 \times 10^{-6}$, color code same as (**B**)), (**F**) 10 Arabic speakers ($n = 0.61$, $\rho = 0.36$, $p = 4.7 \times 10^{-2}$, color code same as (**C**)). Particle emission rate/concentration for saying /ɑ/ at four different levels of loudness by (**G**) 10 Spanish speakers ($n = 0.80$, $\rho = 0.70$, $p = 5.1 \times 10^{-7}$, color code same as (**A**)), (**H**) 10 Mandarin Speakers ($n = 0.85$, $\rho = 0.63$, $p = 1.2 \times 10^{-5}$, color code same as (**B**)), (**I**) 10 Arabic speakers ($n = 0.88$, $\rho = 0.44$, $p = 4.8 \times 10^{-3}$, color code same as (**C**)).

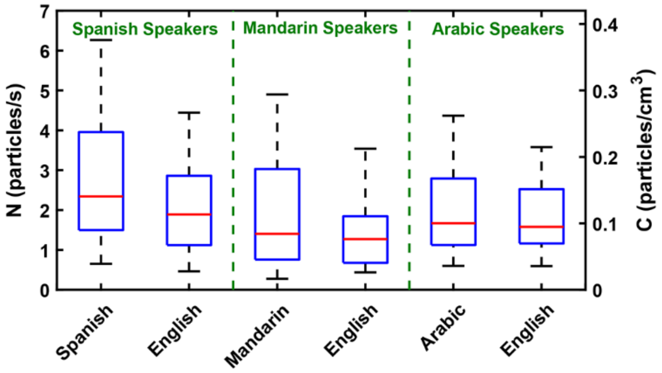

Supplementary Figure S6. Boxplot of particle emission rate/concentration normalized to the loudness amplitude of 0.1 (approximately 85 dB), for thirty bilingual volunteers fluent in both English and either Spanish, Mandarin or Arabic (10 participants each) who read aloud two translations of chapter 24 of "The Little Prince", once in English and once in their other language.

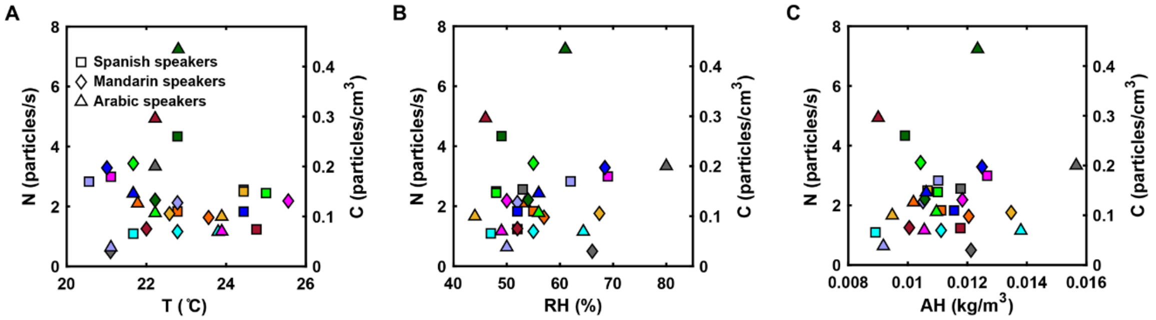

Supplementary Figure S7. Particle emission rate/concentration for reading chapter 24 of "The Little Prince" in English by 30 people (10 Spanish speakers, 10 Mandarin speakers, and 10 Arabic speakers shown with square, diamond, and triangle markers, respectively). The color code for each language speakers is same as Supplementary Fig. S5. Particle emission rate/concentration versus (**A**) temperature, T (ρ = -0.086, and p = 0.652), (**B**) relative humidity, RH (ρ = 0.136, and p = 0.475), and (**C**) absolute humidity, AH (ρ = 0.095, and p = 0.618).

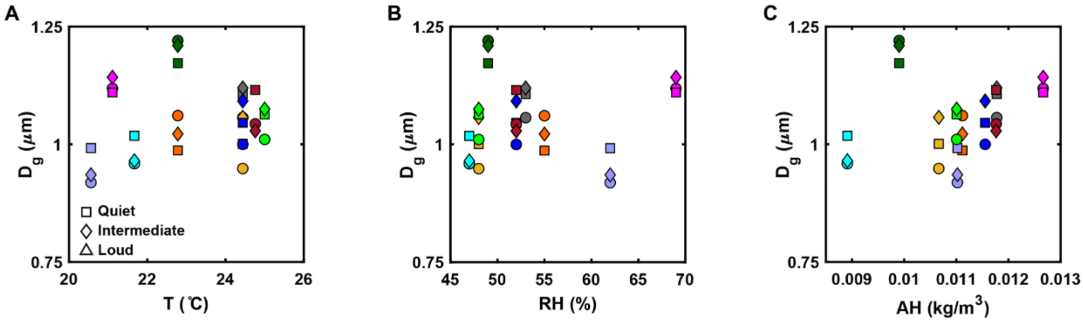

Supplementary Figure S8. Representative measurements of the geometric mean diameter, D_g, versus ambient environmental conditions for reading chapter 24 of "The Little Prince" in English at 3 levels of loudness, by 10 Spanish speakers. Color code is same as Supplementary Fig. S5A. Geometric mean diameter, D_g versus (**A**) temperature, T, (**B**) relative humidity, RH, and (**C**) absolute humidity, AH.

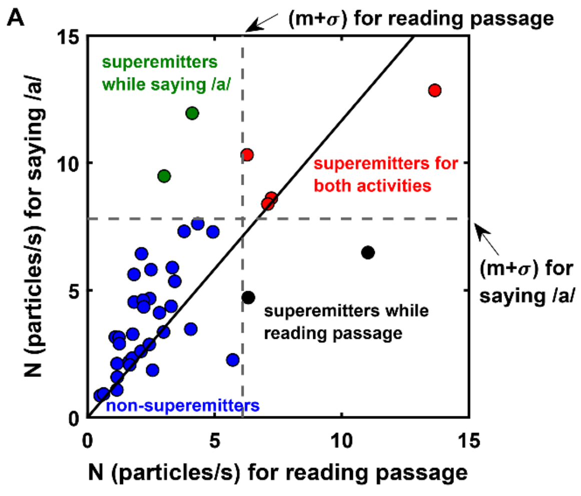

Supplementary Figure S9. Analysis of the superemitters. (**A**) Particle emission rate for saying /ɑ/ vs. reading an English passage at voice amplitude of 0.1 (approximately 85 dB). Blue markers are non-superemitters, and green, black, and red markers are superemitters while saying /ɑ/, while reading passage, and for both activities, respectively. Solid line is the best linear fit (ρ = 0.69, and p = 9.8×10^{-7}). (**B**) Particles emission rate/concentration versus A_{rms}, for two participants, M5 and F4, at three different days. For M5, day 2 and day 3 were, respectively, 4 months and 14 months after day 1; for F4, day 2 and day 3 were, respectively, 12 months and 21 months after day 1.

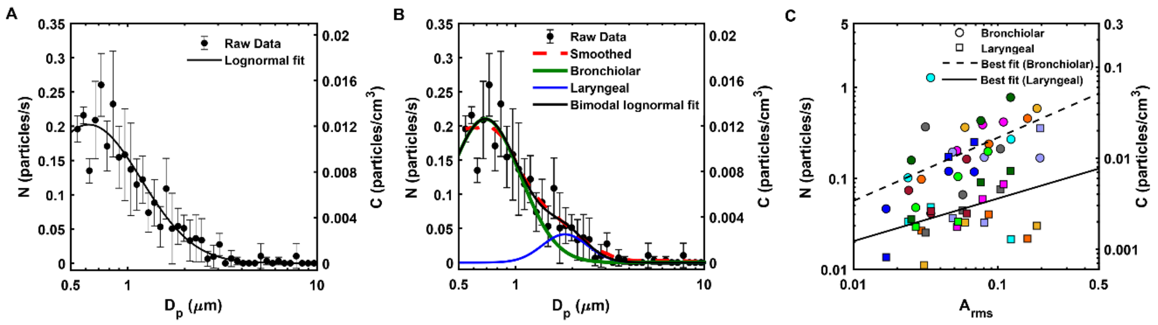

Supplementary Figure S10. Analysis of the particle size distributions. (**A**) Representative particle size distribution for an individual (M3) with unimodal lognormal fit while reading “Rainbow” passage “Loud”. Error bars are one standard deviation using linearly extrapolated values from “Quiet”, and “Intermediate” experiments to the amplitude of the “Loud” experiment, cf. procedure for Fig. 4. (**B**) The same data as in (**A**), but with a bimodal lognormal fit, regressed against smoothed data. Following Johnson et al. (reference 21 in the manuscript), we denote the first peak centered near 0.8 μm as “Bronchiolar” and the second peak centered near 2μm as “Laryngeal.” (**C**) Comparison of the bronchiolar and laryngeal peak emission rates versus speech amplitude for 10 people reading “Rainbow” passage at three levels of loudness. The color code is same as Supplementary Fig. S1.

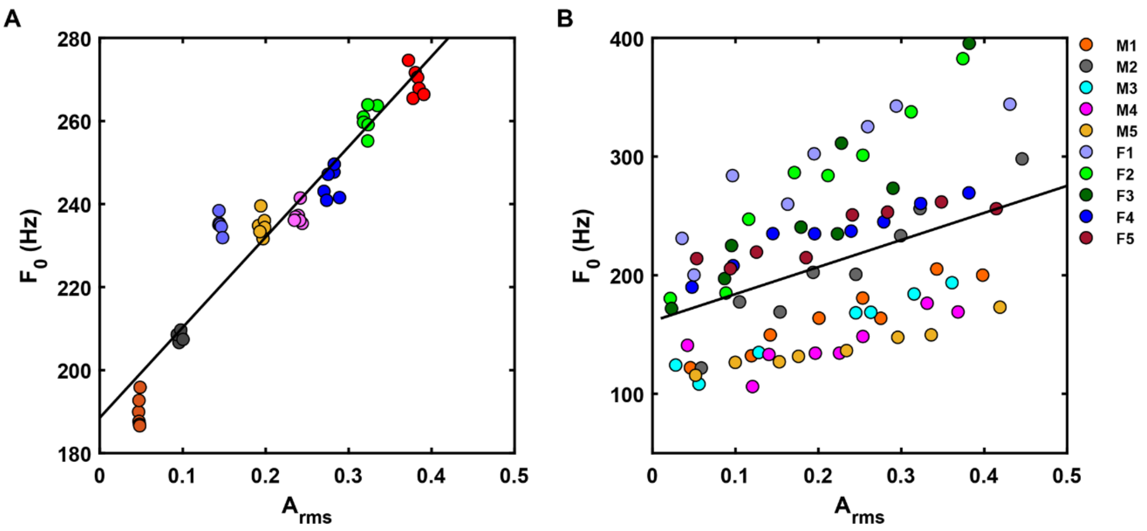

Supplementary Figure S11. (**A**) Fundamental frequency, F_0, versus A_{rms} while saying /ɑ/ for a representative individual participant (F4). Solid line is the best linear fit, with correlation coefficient $\rho = 0.961$ and Pearson's p value = 1.4×10^{-4}. (**B**) Aggregated F_0 vs. A_{rms} data for 10 participants, (5 males, denoted as M1 to M5 and 5 females, denoted as F1 to F5). There are 8 data points for each participant, each representing the average of repeating /ɑ/ six times at approximately the same voice amplitude (cf. Fig. 1). Solid line is the best linear fit, with correlation coefficient $\rho = 0.419$ and Pearson's p value = 1.1×10^{-4}.

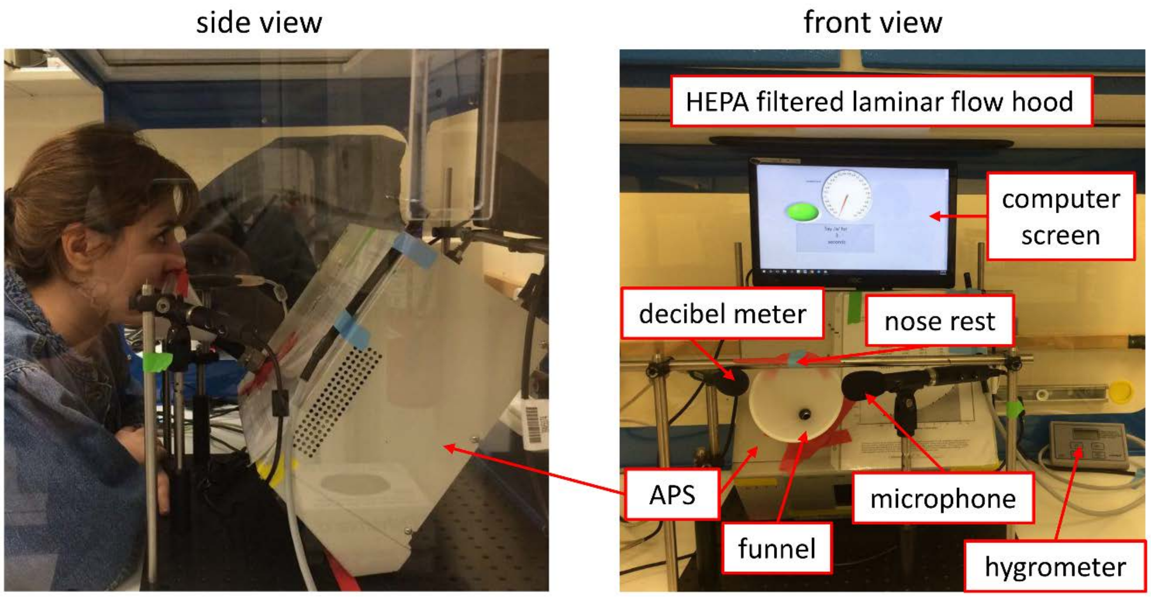

Supplementary Figure S12. Photographs of the experimental apparatus. The subject gave her written informed consent for publication of the image.

Supplementary Table S1. Pairwise comparison p values of breathing/speech experiments, using the Scheffe test, for comparing group means of log-transformed particle emission rate data (p_N), and the corresponding geometric mean diameters (p_{Dg}). Groups with $p<0.05$ are considered significantly different.

**Group 1**	**Group 2**	$\mathbf{p_N}$	$\mathbf{p_{Dg}}$
Nose	Mouth	0.6674	0.9930
Nose	Deep-Fast	0.0010	0.9550
Nose	Fast-Deep	$<10^{-4}$	0.9469
Nose	Quiet	$<10^{-4}$	0.9657
Nose	Intermediate	$<10^{-4}$	0.7671
Nose	Loud	$<10^{-4}$	0.4043
Mouth	Deep-Fast	0.2881	0.6072
Mouth	Fast-Deep	$<10^{-4}$	0.5825
Mouth	Quiet	$<10^{-4}$	1.0000
Mouth	Intermediate	$<10^{-4}$	0.9891
Mouth	Loud	$<10^{-4}$	0.8579
Deep-Fast	Fast-Deep	0.2185	1.0000
Deep-Fast	Quiet	$<10^{-4}$	0.4308
Deep-Fast	Intermediate	$<10^{-4}$	0.1496
Deep-Fast	Loud	$<10^{-4}$	0.0301
Fast-Deep	Quiet	0.1554	0.4070
Fast-Deep	Intermediate	$<10^{-4}$	0.1367
Fast-Deep	Loud	$<10^{-4}$	0.0266
Quiet	Intermediate	0.4166	0.9988
Quiet	Loud	0.0060	0.9459
Intermediate	Loud	0.7814	0.9985
